# On Intermitting Diabetes, and on the Diabetes of Old Age

**Published:** 1853-09

**Authors:** H. B. Jones

**Affiliations:** Physician to St. George’s Hospital


*On Intermitting Diabetes, and on the Diabetes of Old Age.* By H. B. Jones, F.R.S., Physician to St. George's Hospital.—The author's object in this communication was to point out some phenomena connected with diabetes, which he had not found mentioned by other writers. Preliminary to the record of the cases, the author offered some observations on the incorrect results obtained by calculating the amount of sugar present in the urine from the specific gravity. If diabetic urines were solutions of nothing but sugar in distilled water, the tables by Dr. Henry, and the amount of sugar calculated from the specific gravity, would give all the information required; but a multitude of other substances were present besides sugar, each of which was variable, and each of which might cause the specific gravity to vary, whilst the quantity of sugar might remain constant. To be accurate, therefore, the amount of sugar should always be determined by direct experiment, and never calculated from the specific gravity. Results were given, exhibiting the specific gravity, the amount of sugar calculated from solid apparatus, and the absolute amount of sugar obtained by direct analysis. On the subject of intermitting diabetes, the author observed that there could be little doubt that our knowledge of the nature of this disorder might be extended by means of accurate determination of the varieties in the amount of sugar in the urine passed at different periods of the day, and under different circumstances. His object in relation to this form of the disease was to record some cases in which, either from the medical treatment, or the regimen, or the natural course of the complaint, the variation in the amount of sugar was not from much to little, but from highly saccharine urine to total absence of sugar. The state of the urine a few hours after the sugar had disappeared, and an hour or two before it reappeared, was most especially worthy of attention, inasmuch as it might lead to a truer knowledge of the state of the system which preceded the commencement of diabetes. In intermitting diabetes the disease might be seen beginning and ending, and the explanation of the state of the urine which preceded the appearance of the sugar and followed its disappearance, must be included in the true theory of diabetes. Moreover, a better knowledge of the antecedent phenomena might enable us to ward off the disease, if not to treat it with more success. The records of seven cases of the intermitting form of the disease were given, and very minute particulars in several, illustrated the amount of sugar present in the urine at stated intervals in the twenty-four hours, as well as the influence of particular forms of diet on the proportion of sugar excreted. In these cases the state of the urine just after the sugar had disappeared was worthy of attention. A remarkable excess of urea was constantly found before and after the sugar disappeared; and although this might be attributed to the animal diet, yet the occurrence of free uric acid and oxalate of lime in the urine, pointed most clearly to a state of indigestion which was every day to be found without any sugar appearing in the urine. The author offered the following theoretical contrast between ordinary and saccharine indigestion: Ordinary indigestion showed itself in a want of action on the sugar and starch taken as food, in consequence of which excessive acidity was produced—that is, the changes in the non-nitrogenous food were imperfect. Imperfect changes also occurred in the nitrogenous food; this was made evident by an excess of urates and urea in the urine, and perhaps also by the formation of oxalate of lime. In diabetic indigestion the effect might be traced also on the two great classes of food. At first from the non-nitrogenous food sugar was formed instead of acid. Ultimately, if not simultaneously, sometimes the arrest of healthy changes extended to the albuminous food, and, instead of an excess of urates and urea, other products were formed, one of which was sugar. It was possible that some of these products might be found in the urine. Possibly benzoic acid, which is present in some cases of diabetes, in variable quantities, might be one of the new products. Whether this theory were true or not, it was of practical importance to remark the tendency to acidity in these cases of intermitting diabetes. In such cases, animal diet alone, or with alkalies, might stop the formation of sugar. It followed also, that, when oxalate of lime, uric acid, and excess of urea, were found in the urine, it was probable that the diabetes might be temporarily, if not permanently, removed. The occurrence or absence of these substances in the urine might lead to the recognition of the stage of the disease, and they might thus guide us in our prognosis and treatment. The second part of the communication related to the frequency of diabetes in old age. Reference is made to a paper, by M. Dechambre, on this subject, who concluded, from observations made on the urine of old people at the Salpetriere, that sugar was habitually present in the urine of old people. The author gave the particulars of nine cases of diabetes in elderly people, and thought that the occurrence of this affection at the latter periods of life pointed also to the theory of diabetes as an indigestion resulting from an arrest of healthy changes in the food. The cases mentioned in this communication were, in the opinion of the author, opposed to the view of diabetes depending upon an affection of the nerves, or of the liver; and his daily observation led him rather to the view taken by Dr. Prout, that diabetes was an indigestion, and that it first affected the non-nitrogenous, and afterwards the nitrogenous, constituents of our food. As regarded treatment, whatever was beneficial for excessive acidity, was found equally serviceable in diabetes. Alkalies were used in all the cases with benefit. Small meals, free from sugar and acid, and the substances that could give rise to sugar and acids, constituted the best diet. He found, also, that vegetable acids with alkalies were occasionally useful. In a foot-note, the author mentioned some experiments he had not yet published, determining the quantity of sugar in several kinds of beer and wine. Porter contained from 27 to 57 grains of sugar in each ounce of liquid; ale from 43 to 50 grains; beer 25 to 40 grains; port-wine 8.5 to 11 grains; sherry 2 to 4.7 grains; claret none. The absence of all sugar, and the presence of a little alcohol, caused claret to taste highly acid, while the quantity absolutely present was not more, sometimes less, than in other wines which have no acid taste, as, for example, most port wine.—*Medical Times and Gazette.*

